# Supplementary material for: Inhibition of chitin deacetylases to attenuate plant fungal diseases
Source: Nat Commun. 2023 Jun 29;14:3857. doi: 10.1038/s41467-023-39562-7 (PMC10310857; doi:10.1038/s41467-023-39562-7)
Supplement: Supplementary file 1 — Supplementary Information [file 41467_2023_39562_MOESM1_ESM.pdf]

Supplementary Information

Inhibition of chitin deacetylases to attenuate plant fungal diseases

Author names:

Lin Liu[1,2,3,#], Yeqiang Xia[4,5,6,#], Yingchen Li[3,#], Yong Zhou[7], Xiaofeng Su[8], Xiaojing Yan[3], Yan Wang[4,5,6], Wende Liu[3], Hongmei Cheng[8,*], Yuanchao Wang[4,5,6,*], and Qing Yang[1,2,3,*]

Affiliations:

[1]School of Bioengineering, Dalian University of Technology, 116024 Dalian, China.

[2]Shenzhen Branch, Guangdong Laboratory of Lingnan Modern Agriculture, Key Laboratory of Synthetic Biology, Ministry of Agriculture and Rural Affairs, Agricultural Genomics Institute at Shenzhen, Chinese Academy of Agricultural Sciences, 518000 Shenzhen, China.

[3]State Key Laboratory for Biology of Plant Diseases and Insect Pests, Institute of Plant Protection, Chinese Academy of Agricultural Sciences, 100193 Beijing, China.

[4]Department of Plant Pathology, Nanjing Agricultural University, 210095 Nanjing, China.

[5]Key Laboratory of Soybean Disease and Pest Control (Ministry of Agriculture and Rural Affairs), Nanjing Agricultural University, 210095 Nanjing, China.

[6]The Key Laboratory of Plant Immunity, Nanjing Agricultural University, 210095 Nanjing, China.

[7]School of Software, Dalian University of Technology, 116024 Dalian, China.

[8]Biotechnology Research Institute, Chinese Academy of Agricultural Sciences, Beijing, 100081, China.

#These authors contributed equally: Lin Liu, Yeqiang Xia, Yingchen Li.

*Corresponding authors: qingyang@caas.cn (Q. Y.); wangyc@njau.edu.cn (Y. W.); chenghongmei@caas.cn (H. C.).

Table of contents

1. Supplementary Figures

2. Supplementary Tables

1. Supplementary Figures

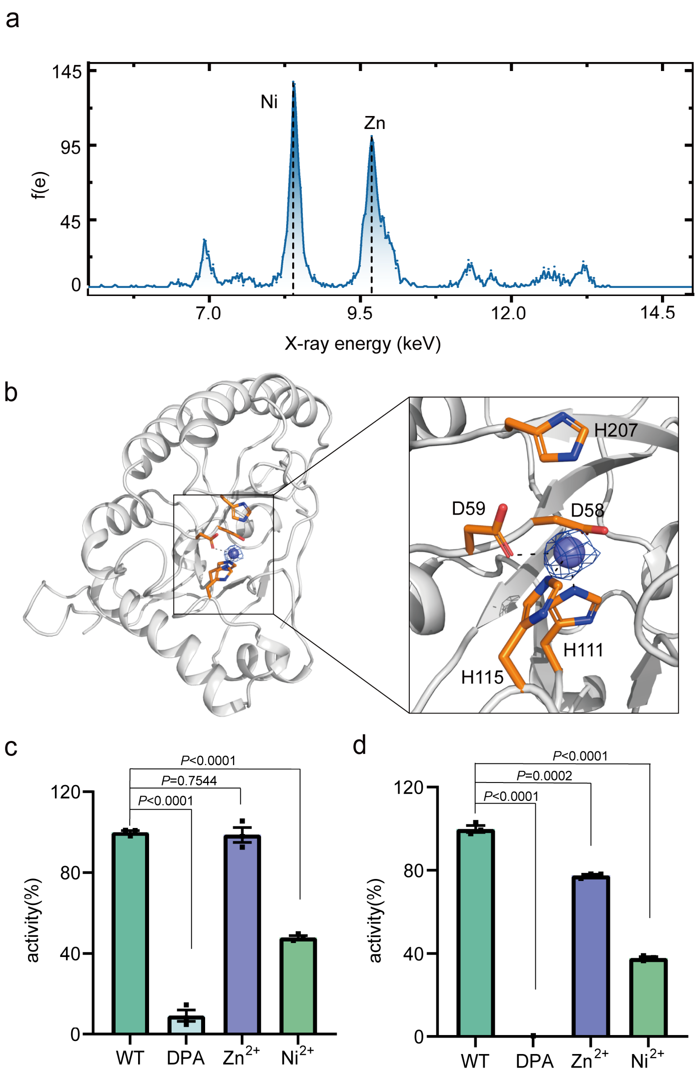

Supplementary Fig. 1| Identification of the metal ion in the active site. a X-ray fluorescence spectrum of a Pst_13661 crystal. **b** Stereo views of anomalous difference density for Zn^{2+} in the structure of Pst_13661. The 2Fo-Fc map around Zn^{2+} is contoured at the 2.0 σ level. **c, d** Relative activity of Pst_13661 (**c**) and *Vd*PDA1 (**d**) with addition of Zn^{2+} and Ni^{2+}. After treatment with 20 mM dipicolinic acid (DPA) to remove the original metal ion, the protein was incubated with 1 mM metal cation ($ZnCl_2$, $NiCl_2$), and the activity was determined with 0.5 mM $(GlcNAc)_3$ as the substrate. Bars in all graphs represent mean values, whiskers represent mean ± SEM, and black circles represent each replicate value (n = 3). Statistical significance was determined by Student's two-sided unpaired *t*-test. *P* values and source data are provided as a Source Data file.

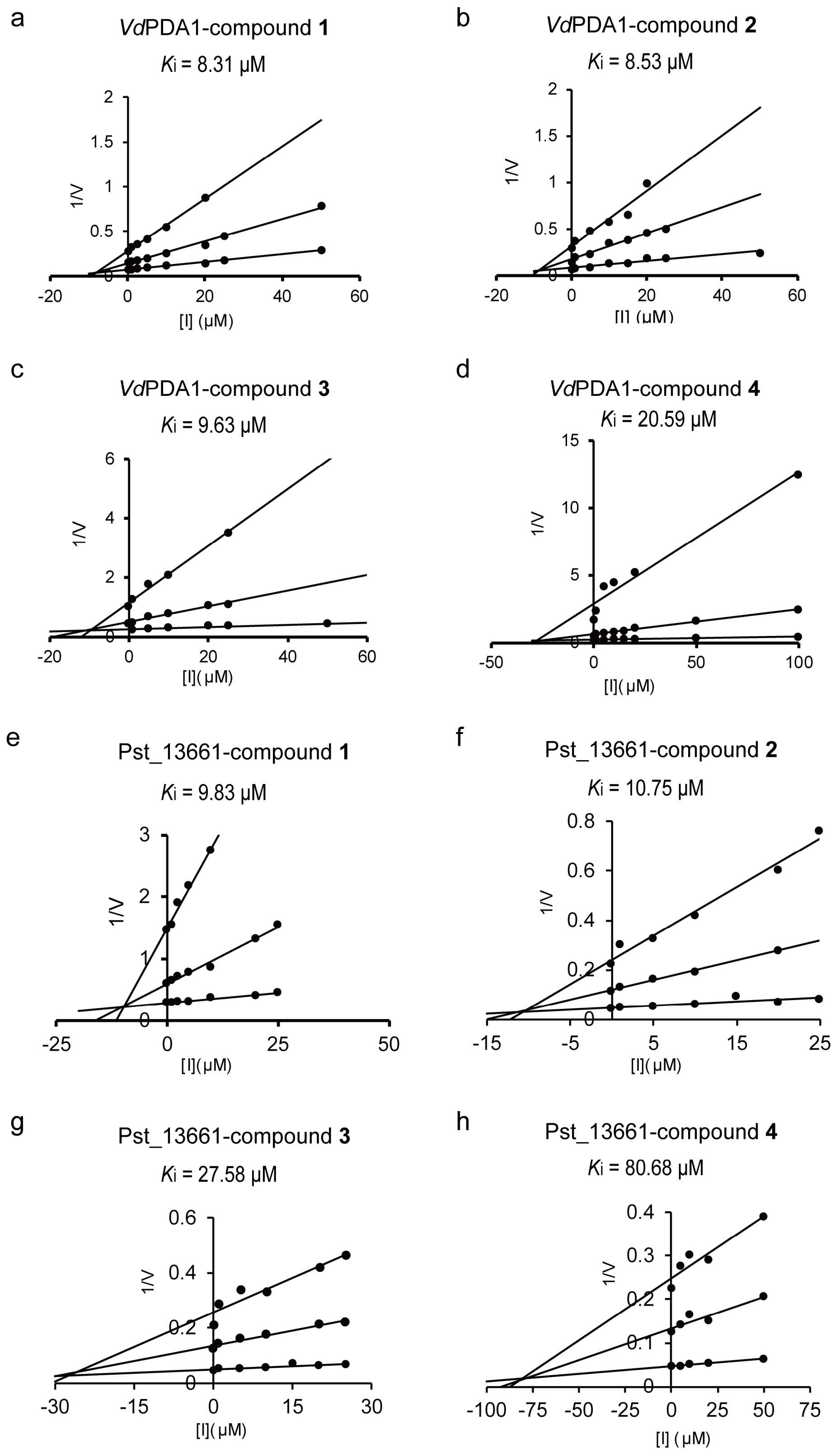

Supplementary Fig. 2| Inhibition constant K_i values of BHA derivatives against *Vd*PDA1 and Pst_13661. a-d The determination of K_i values of compound **1** (**a**), compound **2** (**b**), compound **3** (**c**), compound **4** (**d**) toward *Vd*PDA1; **e-h** K_i values of compound **1** (**e**), compound **2** (**f**), compound **3** (**g**), compound **4** (**h**) toward Pst_13661.

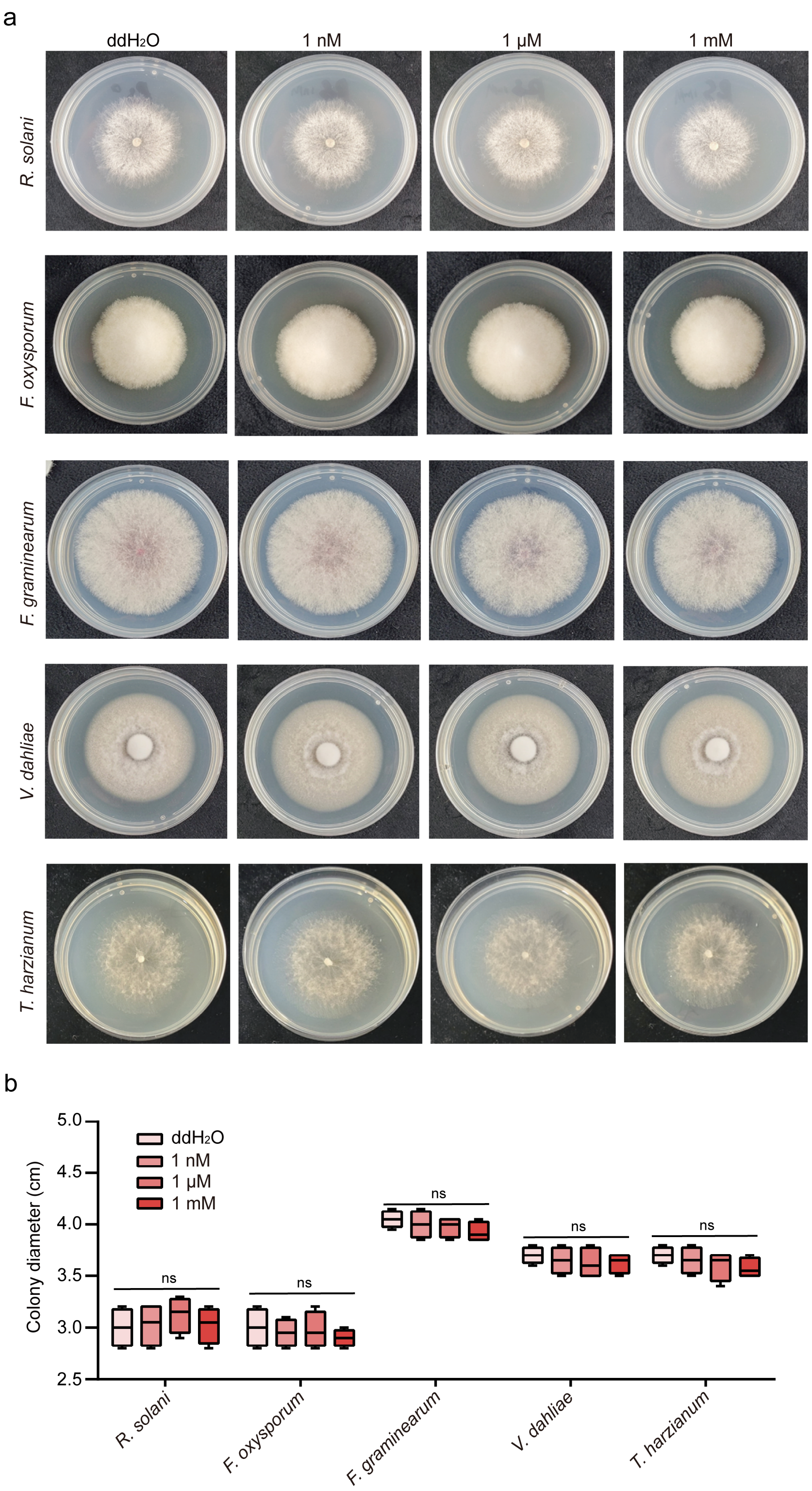

Supplementary Fig. 3| Effects of BHA on fungal mycelial growth. a Colonies of *R. solani*, *F. oxysporum*, *F. graminearum*, *V. dahlia*, and *T. harzianum* on PDA plates. PDA plates were dipped with BHA at different concentrations. **b** Diameter of fungal colonies. The diameter was measured when the fungi grew to about two-thirds of the PDA plate. All experiments were performed in four repeats. No significant difference was found according to Student's two-sided unpaired *t*-test. ns indicates not

significant, $P > 0.05$. Exact P values and source data are provided as a Source Data file. The centre lines are the medians, and the box edges are the 25th and 75th percentiles. The whiskers extend to the minima and maxima within 1.5×the interquartile range (IQR) below and above the lower and upper quartiles, respectively.

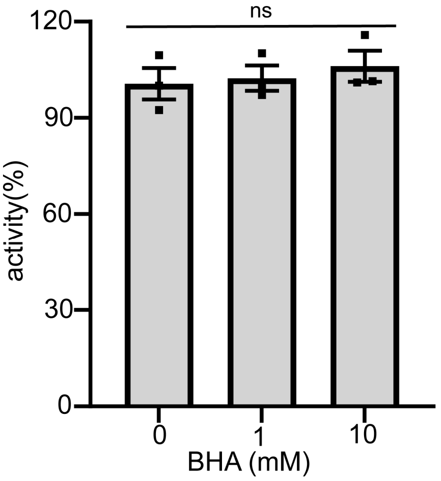

Supplementary Fig. 4| Effect of BHA on the activity of *Bm*CDA7 from the insect *Bombyx mori*. Bars in all graphs represent mean values, whiskers represent mean ± SEM, and black circles represent each replicate value (n = 3). No significant difference was found according to Student's two-sided unpaired *t*-test. ns indicates not significant, $P > 0.05$. Exact *P* values and source data are provided as a Source Data file.

2. Supplementary Tables

Supplementary Table 1. The accession numbers of the fungal CE4 enzymes in the NCBI databases.

Accession number	Species
PNH73948.1	*Verticillium dahliae*
PNH43877.1	*Verticillium dahliae*
XP_009654927.1	*Verticillium dahliae* VdLs.17
KAF3349403.1	*Verticillium dahliae* VDG2
XP_009654342.1	*Verticillium dahliae* VdLs.17
RXG47313.1	*Verticillium dahlia*
RBQ92339.1	*Verticillium dahlia*
KAF3353348.1	*Verticillium dahliae* VDG1
RBQ69250.1	*Verticillium dahlia*
KAF3351784.1	*Verticillium dahliae* VDG2
PNH29399.1	*Verticillium dahlia*
PNH41356.1	*Verticillium dahlia*
XP_009653352.1	*Verticillium dahliae* VdLs.17
KAF3357326.1	*Verticillium dahliae* VDG1
PNH41778.1	*Verticillium dahlia*
XP_009655493.1	*Verticillium dahliae* VdLs.17
XP_003004680.1	*Verticillium alfalfae* VaMs.102
XP_003004379.1	*Verticillium alfalfae* VaMs.102
XP_003002014.1	*Verticillium alfalfae* VaMs.102
XP_003003735.1	*Verticillium alfalfae* VaMs.102
XP_003002224.1	*Verticillium alfalfae* VaMs.102
XP_011324230.1	*Fusarium graminearum* PH-1
CAG1992871.1	*Fusarium graminearum*
PCD27424.1	*Fusarium graminearum*
CAF3501979.1	*Fusarium graminearum*
CAG2010092.1	*Fusarium graminearum*
BAC22123.1	*Fusarium graminearum*
XP_011322165.1	*Fusarium graminearum* PH-1
XP_011326160.1	*Fusarium graminearum* PH-1
CAF3470990.1	*Fusarium graminearum*
EYB32394.1	*Fusarium graminearum*
PCD28042.1	*Fusarium graminearum*
XP_011321484.1	*Fusarium graminearum* PH-1
CAF3511857.1	*Fusarium graminearum*
CZS82386.1	*Fusarium graminearum*
CAG1959665.1	*Fusarium graminearum*
CAG1995104.1	*Fusarium graminearum*
CAF3460628.1	*Fusarium graminearum*
CAF3489571.1	*Fusarium graminearum*

XP_011324448.1	*Fusarium graminearum* PH-1
EYB33928.1	*Fusarium graminearum*
PCD31932.1	*Fusarium graminearum*
KAH7202134.1	*Fusarium oxysporum*
XP_018246303.1	*Fusarium oxysporum* f. sp. *lycopersic*i 4287
EGU87430.1	*Fusarium oxysporum* f. sp. *conglutinans* Fo5176
TVY67972.1	*Fusarium oxysporum* f. sp. *cubense*
TXC02160.1	*Fusarium oxysporum* f. sp. *cubense*
EXA33011.1	*Fusarium oxysporum* f. sp. *pisi* HDV247
SCO90145.1	*Fusarium oxysporum*
XP_031033206.1	*Fusarium oxysporum* NRRL32931
EXA33010.1	*Fusarium oxysporum* f. sp. *pisi*HDV247
ENH73515.1	*Fusarium oxysporum* f. sp. *cubenserace* 1
KAH7205754.1	*Fusarium oxysporum*
RKK12075.1	*Fusarium oxysporum* f. sp. *cepae*
EXL74411.1	*Fusarium oxysporum* f. sp. *conglutinans* race 2 54008
EXM21490.1	*Fusarium oxysporum* f. sp. *vasinfectum* 25433
EWZ29923.1	*Fusarium oxysporum* Fo 47
EXM21489.1	*Fusarium oxysporum* f. sp. *vasinfectum* 25433
XP_018257864.1	*Fusarium oxysporum* f. sp. *lycopersici* 4287
EXL74410.1	*Fusarium oxysporum* f. sp. *conglutinans* race 2 54008
EXK30420.1	*Fusarium oxysporum* f. sp. *melonis* 26406
KAF5263195.1	*Fusarium oxysporum*
RKL30033.1	*Fusarium oxysporum*
KAH7195248.1	*Fusarium oxysporum*
KAG7404961.1	*Fusarium oxysporum* f. sp. *rapae*
EGU88504.1	*Fusarium oxysporum* f. sp. *conglutinans* Fo5176
KAF5254811.1	*Fusarium oxysporum*
KAH7190644.1	*Fusarium oxysporum*
KAG7405961.1	*Fusarium oxysporum* f. sp. *rapae*
EXL40044.1	*Fusarium oxysporum* f. sp. *radicis-lycopersic*i 26381
KAH7216385.1	*Fusarium oxysporum*
EXM15595.1	*Fusarium oxysporum* f. sp. *vasinfectum* 25433
TVY64872.1	*Fusarium oxysporum* f. sp. *cubense*
TXC00211.1	*Fusarium oxysporum* f. sp. *cubense*
KAF5264230.1	*Fusarium oxysporum*
ENH75126.1	*Fusarium oxysporum* f. sp. *cubense* race1
KAH7214768.1	*Fusarium oxysporum*
EXL81763.1	*Fusarium oxysporum* f. sp. *conglutinans* race2 54008
EXM27217.1	*Fusarium oxysporum* f. sp. *vasinfectum* 25433
RKK66337.1	*Fusarium oxysporum*
EXK30146.1	*Fusarium oxysporum* f. sp. *melonis* 26406
XP_031034174.1	*Fusarium oxysporum* NRRL 32931
KAG7407520.1	*Fusarium oxysporum* f. sp. *rapae*

EWZ35707.1	*Fusarium oxysporum* Fo47
PCD29219.1	*Fusarium oxysporum* f. sp. *radicis-cucumerinum*
RYC90809.1	*Fusarium oxysporum* f. sp. *narcissi*
EXA37835.1	*Fusarium oxysporum* f. sp. *pisi* HDV247
SCO89646.1	*Fusarium oxysporum*
EXL55245.1	*Fusarium oxysporum* f. sp. *radicis-lycopersici*26381
KAH7213997.1	*Fusarium oxysporum*
EGU88625.1	*Fusarium oxysporum* f. sp. *conglutinans* Fo5176
RKK46125.1	*Fusarium oxysporum* f. sp. *cepae*
RKK09475.1	*Fusarium oxysporum* f. sp. *cepae*
RKK09653.1	*Fusarium oxysporum* f. sp. *cepae*
EXK77175.1	*Fusarium oxysporum* f. sp. *raphani* 54005
TVY79762.1	*Fusarium oxysporum* f. sp. *cubense*
KAG7420524.1	*Fusarium oxysporum* f. sp. *raphani*
EXL40046.1	*Fusarium oxysporum* f. sp. *radicis-lycopersici* 26381
RKL42969.1	*Fusarium oxysporum*
RYC86268.1	*Fusarium oxysporum* f. sp. *narcissi*
KAH7190642.1	*Fusarium oxysporum*
XP_018249712.1	*Fusarium oxysporum* f. sp. *lycopersici* 4287
SCO82917.1	*Fusarium oxysporum*
KAG7420270.1	*Fusarium oxysporum* f. sp. *rapae*
EXK46105.1	*Fusarium oxysporum* f. sp. *melonis* 26406
EXM34260.1	*Fusarium oxysporum* f. sp. *vasinfectum25433*
SCO82456.1	*Fusarium oxysporum*
ENH71705.1	*Fusarium oxysporum* f. sp. *cubense* race1
EWZ99424.1	*Fusarium oxysporum* f. sp. *lycopersici* MN25
EXL82716.1	*Fusarium oxysporum* f. sp. *conglutinans* race2 54008
QKD50748.1	*Fusarium oxysporum* Fo47
TVY78025.1	*Fusarium oxysporum* f. sp. *cubense*
TXC07372.1	*Fusarium oxysporum* f. sp. *cubense*
RKL31659.1	*Fusarium oxysporum*
EXA48985.1	*Fusarium oxysporum* f. sp. *pisi* HDV247
EWZ48748.1	*Fusarium oxysporum* Fo47
EXL54081.1	*Fusarium oxysporum* f. sp. *radicis-lycopersici* 26381
KAF5257033.1	*Fusarium oxysporum*
EXM24499.1	*Fusarium oxysporum* f. sp. *vasinfectum* 25433
EWZ43705.1	*Fusarium oxysporum* Fo47
RKK76181.1	*Fusarium oxysporum*
KAH7225088.1	*Fusarium oxysporum*
EXA41692.1	*Fusarium oxysporum* f. sp. *pisi* HDV247
KAG6980828.1	*Fusarium oxysporum* f. sp. *conglutinans*
KAH7490006.1	*Fusarium oxysporum* f. sp. *matthiolae*
XP_018235978.1	*Fusarium oxysporum* f. sp. *lycopersici* 4287
ENH69902.1	*Fusarium oxysporum* f. sp. *cubense* race1

KAH7486784.1	*Fusarium oxysporum* f. sp. *matthiolae*
EXL83191.1	*Fusarium oxysporum* f. sp. *conglutinans* race2 54008
XP_031046547.1	*Fusarium oxysporum* NRRL 32931
KAH7229690.1	*Fusarium oxysporum*
RKL18035.1	*Fusarium oxysporum*
EXK41055.1	*Fusarium oxysporum* f. sp. *melonis* 26406
EXK88489.1	*Fusarium oxysporum* f. sp. *raphani* 54005
CUA70172.1	*Rhizoctonia solani*
CAE6478110.1	*Rhizoctonia solani*
XP_043184837.1	*Rhizoctonia solani*
KAF8760068.1	*Rhizoctonia solani*
KAF8711685.1	*Rhizoctonia solani*
KAF8673086.1	*Rhizoctonia solani*
CEL53831.1	*Rhizoctonia solani* AG-1 IB
KAF8673085.1	*Rhizoctonia solani*
KAF8745434.1	*Rhizoctonia solani*
KAF8681313.1	*Rhizoctonia solani*
CAE6343028.1	*Rhizoctonia solani*
CUA78459.1	*Rhizoctonia solani*
KAF8756197.1	*Rhizoctonia solani*
CCO34409.1	*Rhizoctonia solani* AG-1 IB
CAE6447031.1	*Rhizoctonia solani*
CAE6435325.1	*Rhizoctonia solani*
CAE6469351.1	*Rhizoctonia solani*
XP_043176289.1	*Rhizoctonia solani*
KAF8711684.1	*Rhizoctonia solani*
CAE6401881.1	*Rhizoctonia solani*
CAE6421252.1	*Rhizoctonia solani*
CAE7054025.1	*Rhizoctonia solani*
CAE6447680.1	*Rhizoctonia solani*
KAH7334436.1	*Rhizoctonia solani*
KDN35791.1	*Rhizoctonia solani* AG-8 WAC10335
CCO26450.1	*Rhizoctonia solani* AG-1 IB
CUA77894.1	*Rhizoctonia solani*
CAE6413053.1	*Rhizoctonia solani*
KAH7334434.1	*Rhizoctonia solani*
KAF8709516.1	*Rhizoctonia solani*
CAE6523299.1	*Rhizoctonia solani*
EUC63918.1	*Rhizoctonia solani* AG-3 Rhs1AP
KEP47598.1	*Rhizoctonia solani* 123E
CAE6447671.1	*Rhizoctonia solani*
CAE6477776.1	*Rhizoctonia solani*
KAF8683313.1	*Rhizoctonia solani*
CAE6427622.1	*Rhizoctonia solani*

CEL57067.1	*Rhizoctonia solani* AG-1 IB
CUA77879.1	*Rhizoctonia solani*
CAE6489829.1	*Rhizoctonia solani*
KEP50006.1	*Rhizoctonia solani* 123E
CAE6420383.1	*Rhizoctonia solani*
CAE6437032.1	*Rhizoctonia solani*
CAE6452667.1	*Rhizoctonia solani*
KAH7334435.1	*Rhizoctonia solani*
CAE6489819.1	*Rhizoctonia solani*
CUA73215.1	*Rhizoctonia solani*
XP_043180094.1	*Rhizoctonia solani*
CAE7229117.1	*Rhizoctonia solani*
CUA77895.1	*Rhizoctonia solani*
CAE6434365.1	*Rhizoctonia solani*
CAE6447338.1	*Rhizoctonia solani*
CAE6428034.1	*Rhizoctonia solani*
CEL63414.1	*Rhizoctonia solani* AG-1 IB
CUA74802.1	*Rhizoctonia solani*
KAF8669757.1	*Rhizoctonia solani*
EUC54381.1	*Rhizoctonia solani* AG-3 Rhs1AP
KAF8693892.1	*Rhizoctonia* solani
CAE6401893.1	*Rhizoctonia solani*
CAE6418474.1	*Rhizoctonia solani*
CAE6396109.1	*Rhizoctonia solani*
CAE6445168.1	*Rhizoctonia solani*
EUC57794.1	*Rhizoctonia solani* AG-3 Rhs1AP
CAE6502756.1	*Rhizoctonia solani*
KAH7337323.1	*Rhizoctonia solani*
CAE6420388.1	*Rhizoctonia solani*
XP_043184842.1	*Rhizoctonia solani*
CAE6455340.1	*Rhizoctonia solani*
XP_043185103.1	*Rhizoctonia solani*
CUA77896.1	*Rhizoctonia solani*
CAE6440308.1	*Rhizoctonia solani*
CAE6418414.1	*Rhizoctonia solani*
KDN34895.1	*Rhizoctonia solani* AG-8 WAC10335
CUA78404.1	*Rhizoctonia solani*
KAF8709513.1	*Rhizoctonia solani*
CAE6441903.1	*Rhizoctonia solani*
CAE7097180.1	*Rhizoctonia solani*
CAE6478135.1	*Rhizoctonia solani*
EUC57793.1	*Rhizoctonia solani* AG-3 Rhs1AP
KAH7339072.1	*Rhizoctonia solani*
CAE6455348.1	*Rhizoctonia solani*

CAE6440302.1	*Rhizoctonia solani*
KAF8681051.1	*Rhizoctonia solani*
KAF8760069.1	*Rhizoctonia solani*
KAH7334648.1	*Rhizoctonia solani*
CAE7054065.1	*Rhizoctonia solani*
KAF8706912.1	*Rhizoctonia solani*
EUC60397.1	*Rhizoctonia solani* AG-3 Rhs1AP
KAF8678954.1	*Rhizoctonia solani*
CAE6444900.1	*Rhizoctonia solani*
CUA66943.1	*Rhizoctonia solani*
KEP47932.1	*Rhizoctonia solani* 123E
CAE6449324.1	*Rhizoctonia solani*
CAE6413064.1	*Rhizoctonia solani*
CCO26451.1	*Rhizoctonia solani* AG-1 IB
CAE6526854.1	*Rhizoctonia solani*
KDN34466.1	*Rhizoctonia solani* AG-8 WAC10335
KAH7027082.1	*Macrophomina phaseolina*
EKG19456.1	*Macrophomina phaseolina* MS6
KAH7042126.1	*Macrophomina phaseolina*
EKG20639.1	*Macrophomina phaseolina* MS6
KAH7027096.1	*Macrophomina phaseolina*
EKG19445.1	*Macrophomina phaseolina* MS6
EKG14539.1	*Macrophomina phaseolina* MS6
KAH7062516.1	*Macrophomina phaseolina*
KAF4041905.1	*Phytophthora infestans*
XP_002903952.1	*Phytophthora infestans* T30-4
XP_009524771.1	*Phytophthora sojae*
POM57390.1	*Phytophthora palmivora* var. palmivora
POM80773.1	*Phytophthora palmivora* var. palmivora
XP_003719393.1	*Pyricularia oryzae* 70-15
XP_003712529.1	*Pyricularia oryzae* 70-15
XP_003715704.1	*Pyricularia oryzae* 70-15
XP_003714889.1	*Pyricularia oryzae* 70-15
QBZ56729.1	*Pyricularia oryzae*
ELQ41555.1	*Pyricularia oryzae* Y34
QBZ59347.1	*Pyricularia oryzae*
ELQ37478.1	*Pyricularia oryzae* Y34
XP_016845950.1	*Pyricularia oryzae* 70-15
BAI44124.1	*Pyricularia oryzae*
QBZ65515.1	*Pyricularia oryzae*
XP_003712634.1	*Pyricularia oryzae* 70-15
QBZ64097.1	*Pyricularia oryzae*
XP_003719200.1	*Pyricularia oryzae* 70-15
ELQ40322.1	*Pyricularia oryzae* Y34

XP_003716465.1	*Pyricularia oryzae* 70-15
QBZ59453.1	*Pyricularia oryzae*
ELQ39280.1	*Pyricularia oryzae* Y34
ELQ62073.1	*Pyricularia oryzae* P131
KNE92947.1	*Puccinia striiformis* f. sp. *tritici* PST-78
KNE92929.1	*Puccinia striiformis* f. sp. *tritici* PST-78
KNF00609.1	*Puccinia striiformis* f. sp. *tritici* PST-78
KNE92928.1	*Puccinia striiformis* f. sp. *tritici* PST-78
KNF05358.1	*Puccinia striiformis* f. sp. *tritici* PST-78
KNF00610.1	*Puccinia striiformis* f. sp. *tritici* PST-78
KNE92948.1	*Puccinia striiformis* f. sp. *tritici* PST-78
KNE99430.1	*Puccinia striiformis* f. sp. *tritici* PST-78
KNF00443.1	*Puccinia striiformis* f. sp. *tritici* PST-78
KNF00442.1	*Puccinia striiformis* f. sp. *tritici* PST-78
KNF00448.1	*Puccinia striiformis* f. sp. *tritici* PST-78
KNE95266.1	*Puccinia striiformis* f. sp. *tritici* PST-78
KNF03934.1	*Puccinia* stri*i*formis f. sp. *tritici* PST-78
KAA1119399.1	*Puccinia graminis* f. sp. *tritici*
KAA1066893.1	*Puccinia graminis* f. sp. *tritici*
XP_003322955.2	*Puccinia graminis* f. sp. *tritici* CRL 75-36-700-3
KAA1085342.1	*Puccinia graminis* f. sp. *tritici*
XP_003329508.2	*Puccinia graminis* f. sp. *tritici* CRL75-36-700-3
KAA1083983.1	*Puccinia graminis* f. sp. *tritici*
XP_003320832.2	*Puccinia graminis* f. sp. *tritici* CRL 75-36-700-3
KAA1118438.1	*Puccinia graminis* f. sp. *tritici*
KAA1128461.1	*Puccinia graminis* f. sp. *tritici*
KAA1082627.1	*Puccinia graminis* f. sp. *tritici*
KAA1079405.1	*Puccinia graminis* f. sp. *tritici*
KAA1068537.1	*Puccinia graminis* f. sp. *tritici*
XP_003327442.2	*Puccinia graminis* f. sp. *tritici* CRL 75-36-700-3
KAA1069419.1	*Puccinia graminis* f. sp. *tritici*
KAA1111304.1	*Puccinia graminis* f. sp. *tritici*
KAA1085341.1	*Puccinia graminis* f. sp. *tritici*
KAA1082120.1	*Puccinia graminis* f. sp. *tritici*
XP_003329509.2	*Puccinia graminis* f. sp. *tritici* CRL75-36-700-3
XP_003323413.1	*Puccinia graminis* f. sp. *tritici* CRL 75-36-700-3
KAA1128669.1	*Puccinia graminis* f. sp. *tritici*
KAA1065378.1	*Puccinia graminis* f. sp. *tritici*
XP_003338851.2	*Puccinia graminis* f. sp. *tritici* CRL 75-36-700-3
KAA1110256.1	*Puccinia graminis* f. sp. *tritici*
XP_003322147.2	*Puccinia graminis* f. sp. *tritici* CRL75-36-700-3
KAA1085572.1	*Puccinia graminis* f. sp. *tritici*
XP_003328341.2	*Puccinia graminis* f. sp. *tritici* CRL 75-36-700-3
KAA1074520.1	*Puccinia graminis* f. sp. *tritici*

XP_003336703.1	*Puccinia graminis* f. sp. *tritici* CRL 75-36-700-3
XP_003329836.1	*Puccinia graminis* f. sp. *tritici* CRL 75-36-700-3
KAA1080497.1	*Puccinia graminis* f. sp. *tritici*
KAA1112743.1	*Puccinia graminis* f. sp. *tritici*
KAA1088146.1	*Puccinia graminis* f. sp. *tritici*
KAA1096223.1	*Puccinia graminis* f. sp. *tritici*
KAA1099486.1	*Puccinia graminis* f. sp. *tritici*
KAA1117479.1	*Puccinia graminis* f. sp. *tritici*
KAA1120566.1	*Puccinia graminis* f. sp. *tritici*
XP_003326034.2	*Puccinia graminis* f. sp. *tritici* CRL 75-36-700-3
KAA1110014.1	*Puccinia graminis* f. sp. *tritici*
KAA1110013.1	*Puccinia graminis* f. sp. *tritici*
KAA1074229.1	*Puccinia graminis* f. sp. *tritici*
KAA1074230.1	*Puccinia graminis* f. sp. *tritici*
KAA1100527.1	*Puccinia graminis* f. sp. *tritici*
XP_003325268.2	*Puccinia graminis* f. sp. *tritici* CRL 75-36-700-3
Q6	DWK3.1 *Colletotrichum lindemuthianum*
KAF4869354.1	*Colletotrichum siamense*
KAF4828987.1	*Colletotrichum tropicale*
KAF4810982.1	*Colletotrichum siamense*
XP_037173769.1	*Colletotrichum aenigma*
KDN69886.1	*Colletotrichum sublineola*
XP_036490329.1	*Colletotrichum siamense*
EQB59400.1	*Colletotrichum gloeosporioides* Cg-14
KAF4892828.1	*Colletotrichum viniferum*
KAF3797152.1	*Colletotrichum gloeosporioides*
KAF4842325.1	*Colletotrichum siamense*
XP_031892338.1	*Colletotrichum fructicola*
KAF0329877.1	*Colletotrichum asianum*
TDZ61679.1	*Colletotrichum trifolii*
TDZ35904.1	*Colletotrichum spinosum*
TEA19173.1	*Colletotrichum sidae*
XP_036589105.1	*Colletotrichum truncatum*
KAF6834231.1	*Colletotrichum plurivorum*
TDZ21020.1	*Colletotrichum orbiculareMAFF240422*
KXH57867.1	*Colletotrichum salicis*
KAF6807766.1	*Colletotrichum sojae*
OLN96571.1	*Colletotrichum chlorophyti*
KAH0420507.1	*Colletotrichum camelliae*
XP_038748947.1	*Colletotrichum karsti*
KXH27886.1	*Colletotrichum nymphaeaeSA*-01
XP_008096791.1	*Colletotrichum graminicola* M1.001
XP_018157093.1	*Colletotrichum higginsianum* IMI 349063
TIC95269.1	*Colletotrichum higginsianum*

KZL83893.1	*Colletotrichum incanum*
KAG7058535.1	*Colletotrichum scovillei*
KZL75874.1	*Colletotrichum tofieldiae*
KXH44856.1	*Colletotrichum simmondsii*
EXF82578.1	*Colletotrichum fioriniae* PJ7
XP_035332001.1	*Colletotrichum scovillei*
TQN65200.1	*Colletotrichum shisoi*
KAF6808319.1	*Colletotrichum musicola*
TKW53251.1	*Colletotrichum tanaceti*
XP_036582700.1	*Colletotrichum truncatum*
TDZ71838.1	*Colletotrichum trifolii*
TDZ18517.1	*Colletotrichum orbiculare* MAFF 240422
TDZ37263.1	*Colletotrichum spinosum*
CAQ16203.1	*Colletotrichum graminicola*
KAF4923742.1	*Colletotrichum fructicola*
KAF4816428.1	*Colletotrichum siamense*
XP_036497440.1	*Colletotrichum siamense*
KAF4871841.1	*Colletotrichum siamense*
EQB47605.1	*Colletotrichum gloeosporioides* Cg-14
XP_031878871.1	*Colletotrichum fructicola*
KAF3802619.1	*Colletotrichum gloeosporioides*
KAF4905512.1	*Colletotrichum viniferum*
KDN72346.1	*Colletotrichum sublineola*
XP_018156777.1	*Colletotrichum higginsianum* IMI 349063
KAH0441198.1	*Colletotrichum camelliae*
KAF4856769.1	*Colletotrichum siamense*
XP_037182128.1	*Colletotrichum aenigma*
KAF4834855.1	*Colletotrichum tropicale*
KAF0331972.1	*Colletotrichum asianum*
TQN64709.1	*Colletotrichum shisoi*
TKW52433.1	*Colletotrichum tanaceti*
TEA14565.1	*Colletotrichum sidae*
TDZ36131.1	*Colletotrichum spinosum*
TDZ73470.1	*Colletotrichum trifolii*
TDZ18917.1	*Colletotrichum orbiculare* MAFF240422
XP_031891222.1	*Colletotrichum fructicola*
TID02824.1	*Colletotrichum higginsianum*
TKW50760.1	*Colletotrichum tanaceti*
KAF4887872.1	*Colletotrichum fructicola*
KAF4490962.1	*Colletotrichum fructicola* Naragc 5
KAF6834210.1	*Colletotrichum musicola*
KXH36770.1	*Colletotrichum salicis*
KAF6801221.1	*Colletotrichum sojae*
XP_018161804.1	*Colletotrichum higginsianum* IMI 349063

KAF4424928.1	*Colletotrichum fructicola*
KAF0328503.1	*Colletotrichum asianum*
KAF3797887.1	*Colletotrichum gloeosporioides*
KAF4807867.1	*Colletotrichum siamense*
KAF4831225.1	*Colletotrichum tropicale*
XP_037185119.1	*Colletotrichum aenigma*
EQB54867.1	*Colletotrichum gloeosporioides* Cg-14
KAF4848430.1	*Colletotrichum siamense*
KAF4923096.1	*Colletotrichum viniferum*
XP_035327926.1	*Colletotrichum scovillei*
XP_036502802.1	*Colletotrichum siamense*
KXH41075.1	*Colletotrichum nymphaeae* SA-01
EXF84202.1	*Colletotrichum fioriniae* PJ7
XP_038747517.1	*Colletotrichum karsti*
KXH44862.1	*Colletotrichum nymphaeae* SA-01
TQN69797.1	*Colletotrichum shisoi*
XP_036578724.1	*Colletotrichum truncatum*
KDN61315.1	*Colletotrichum sublineola*
KZL75984.1	*Colletotrichum tofieldiae*
XP_035327733.1	*Colletotrichum scovillei*
XP_038747331.1	*Colletotrichum karsti*
TEA19991.1	*Colletotrichum sidae*
KXH28459.1	*Colletotrichum salicis*
EXF85482.1	*Colletotrichum fioriniae* PJ7
TDZ18932.1	*Colletotrichum orbiculare* MAFF 240422
TDZ29148.1	*Colletotrichum spinosum*

Supplementary Table 2. The primers used in this study.

Primer name	Sequence	Illustration
GhMPK6-QF	AGGTCACTGCTAAATATAAACCAC	qRT-PCR
GhMPK6-QR	TTCTCTTCGCATCAATCTTGTTAT	qRT-PCR
GbRboh5/18-QF	GACTTGACCGCCTCTACGAAT	qRT-PCR
GbRboh5/18-QR	CTGACTTCTTCTTCTGTTATTCTTCC	qRT-PCR
Ghactin-QF	TCCCATTGAGCATGGGATCG	qRT-PCR
Ghactin-QR	CGTGAGAAGAACAGGGTGC	qRT-PCR
Rs-actin-QF	CCAACCGAGAAAAGATGACGC	qRT-PCR
Rs-actin-QR	CGTAAATTGGAACCGTATGCG	qRT-PCR
Fo-actin-QF	CCGAGGCTCCCATCAACC	qRT-PCR
Fo-actin-QR	GGCGAAACCCTCGTAAATGG	qRT-PCR
Fg-actin-QF:	GTCCAATCCACTCCATCCTC	qRT-PCR
Fg-actin-QR:	CGGTCTTCTCGAGAGGTTCA	qRT-PCR
GmCYP-actin-QF	CGGGACCAGTGTGCTTCTTCA	qRT-PCR
GmCYP-actin-QR	CCCCTCCACTACAAAGGCTCG	qRT-PCR
VdPAD1-UF1	GGTCTTAAUGCCACGCGCATGACGCAC	*VdPDA1* deletion
*VdPAD*1-UR1	GGTCTTAAUGCCACGCGCATGACGCAC	*VdPDA1* deletion
VdPAD1-DF2	GGACTTAAUATGTCCTCATCGAGGGCC	*VdPDA1* deletion
VdPAD1-DR2	GGGTTTAAUCGGACGATTGCGAATGGTTC	*VdPDA1* deletion
VdPAD1-F	acccaagcatcgatcccgggATGTTTGTCAACCTCCGCAA	complementary mutant
VdPAD1-R	atggtaccgtcgaccccgggAGCGCGGGGAGTGACCCT	complementary mutant
KO*VdPDA1*-UF	GTCAGCCGAGGCCTTTCCTCCTACC	knockout mutant sequencing
KO*VdPDA1*-DR	TGGGACCTTGAGGATGACTGGTTTG	knockout mutant sequencing
VdPDA1-F	CATTTGGACCGAGAACGCCGTC	verification primer
VdPDA1-R	CCTCTGGACCTCCTTGATCATGGC	verification primer
