# Supplementary material for: Inhibition of chitin deacetylases to attenuate plant fungal diseases
Source: Nat Commun. 2023 Jun 29;14:3857. doi: 10.1038/s41467-023-39562-7 (PMC10310857; doi:10.1038/s41467-023-39562-7)
Supplement: Supplementary file 2 — Reporting Summary [file 41467_2023_39562_MOESM2_ESM.pdf]

nature portfolio

Corresponding author(s): Qing Yang

Last updated by author(s): May 28, 2023

Reporting Summary

Nature Portfolio wishes to improve the reproducibility of the work that we publish. This form provides structure for consistency and transparency in reporting. For further information on Nature Portfolio policies, see our Editorial Policies and the Editorial Policy Checklist.

Statistics

For all statistical analyses, confirm that the following items are present in the figure legend, table legend, main text, or Methods section.

n/a	Confirmed	
☐	☒	The exact sample size (*n*) for each experimental group/condition, given as a discrete number and unit of measurement
☐	☒	A statement on whether measurements were taken from distinct samples or whether the same sample was measured repeatedly
☐	☒	The statistical test(s) used AND whether they are one- or two-sided<br>*Only common tests should be described solely by name; describe more complex techniques in the Methods section.*
☒	☐	A description of all covariates tested
☒	☐	A description of any assumptions or corrections, such as tests of normality and adjustment for multiple comparisons
☐	☒	A full description of the statistical parameters including central tendency (e.g. means) or other basic estimates (e.g. regression coefficient) AND variation (e.g. standard deviation) or associated estimates of uncertainty (e.g. confidence intervals)
☐	☒	For null hypothesis testing, the test statistic (e.g. *F*, *t*, *r*) with confidence intervals, effect sizes, degrees of freedom and *P* value noted<br>*Give P values as exact values whenever suitable.*
☒	☐	For Bayesian analysis, information on the choice of priors and Markov chain Monte Carlo settings
☒	☐	For hierarchical and complex designs, identification of the appropriate level for tests and full reporting of outcomes
☒	☐	Estimates of effect sizes (e.g. Cohen's *d*, Pearson's *r*), indicating how they were calculated

Our web collection on statistics for biologists contains articles on many of the points above.

Software and code

Policy information about availability of computer code

Data collection	Blu-Ice BL18U1, BL19U1, BL17B1, AutoEmation
Data analysis	UCSF ChimeraX 1.4, Coot 0.9.4.1, PHENIX 1.19.2-4158, PyMOL 2.5.1, GraphPad Prism 9.4.1, OriginPro 8.5, HKL3000v721.3, PROCHECK [https://www.ebi.ac.uk/thornton-srv/software/PROCHECK/index.html], ClustalW [www.ebi.ac.uk/clustalw/], MEGA 3.0, Phaser 2.6.0, Excel 365

For manuscripts utilizing custom algorithms or software that are central to the research but not yet described in published literature, software must be made available to editors and reviewers. We strongly encourage code deposition in a community repository (e.g. GitHub). See the Nature Portfolio guidelines for submitting code & software for further information.

Data

Policy information about availability of data

All manuscripts must include a data availability statement. This statement should provide the following information, where applicable:

- Accession codes, unique identifiers, or web links for publicly available datasets
- A description of any restrictions on data availability
- For clinical datasets or third party data, please ensure that the statement adheres to our policy

All data generated in this study are available in the main text, supplementary materials, or the source data file except for the structural data that have been deposited to the Protein Data Bank [https://www.rcsb.org] under the accession code 8HFA (VdPDA1) [https://doi.org/10.2210/pdb8HFA/pdb], 8HF9 (Pst_13661) [https://doi.org/10.2210/pdb8HF9/pdb], 8HE1 (Pst_13661-BHA) [https://doi.org/10.2210/pdb8HE1/pdb], 8HE2 (Pst_13661-compound 2) [https://doi.org/10.2210/

pdb8HE2/pdb], 8HE4 (Pst_13661-compound 3) [https://doi.org/10.2210/pdb8HE4/pdb]. The structure model for molecular replacement used in this study are available in the Protein Data Bank under accession code 2IW0 [https://www.pdbus.org/structure/2IW0]. The sequences used in this study to generate phylogenetic tree are available in the NCBI database [https://www.ncbi.nlm.nih.gov/] under accession codes that are presented in Supplementary Table 1. Source data are provided with this paper.

Human research participants

Policy information about studies involving human research participants and Sex and Gender in Research.

Reporting on sex and gender	N/A
Population characteristics	N/A
Recruitment	N/A
Ethics oversight	N/A

Note that full information on the approval of the study protocol must also be provided in the manuscript.

Field-specific reporting

Please select the one below that is the best fit for your research. If you are not sure, read the appropriate sections before making your selection.

- [x] Life sciences
- [ ] Behavioural & social sciences
- [ ] Ecological, evolutionary & environmental sciences

For a reference copy of the document with all sections, see nature.com/documents/nr-reporting-summary-flat.pdf

Life sciences study design

All studies must disclose on these points even when the disclosure is negative.

Sample size	The sample sizes were reasonable numbers for the statistical analysis using in this paper. Sample sizes for pathogenicity and defense response gene expression on plants were nine (three technical repeats with three biological repeats), and for colony diameter assays were four plates per difference concentration BHA treatment. The sample sizes were described in the figure legends.
Data exclusions	No data have been excluded.
Replication	Each experiments were repeated independently at least three times, with all tempts are successful.
Randomization	The experiment design and the experiment data collection are randomized. For each independent experiment, the plants (including soybean seedlings, cotton seedings and wheat seedlings) which used in this paper were selected under identical experimental conditions and randomly assigned to treatment and control groups.
Blinding	The infection assays were recorded in a blind way.

Reporting for specific materials, systems and methods

We require information from authors about some types of materials, experimental systems and methods used in many studies. Here, indicate whether each material, system or method listed is relevant to your study. If you are not sure if a list item applies to your research, read the appropriate section before selecting a response.

Materials & experimental systems

n/a	Involved in the study	
x		Antibodies
	x	Eukaryotic cell lines
x		Palaeontology and archaeology
x		Animals and other organisms
x		Clinical data
x		Dual use research of concern

Methods

n/a	Involved in the study	
x		ChIP-seq
x		Flow cytometry
x		MRI-based neuroimaging

Eukaryotic cell lines

Policy information about cell lines and Sex and Gender in Research

Cell line source(s)	Pichia pastoris GS115 (Invitrogen), validation reports are available on the supplier website (https://www.thermofisher.cn/order/catalog/product/C18100).
Authentication	No further authentication was performed for commercially available cell lines.
Mycoplasma contamination	Not tested for mycoplasma contamination.
Commonly misidentified lines (See ICLAC register)	No commonly misidentified cell lines were used.
